# An NMR-Based Metabolic Signature to Identify Clinically Significant Prostate Cancer in Patients Undergoing Biopsy

**DOI:** 10.1210/clinem/dgae704

**Published:** 2024-10-09

**Authors:** Michael Ladurner, Tobias Ameismeier, Helmut Klocker, Eberhard Steiner, Helga Hauffe, Gerhard P Aigner, Hannes Neuwirt, Tina Böld, Selina Strathmeyer, Isabel Heidegger, Diana Drettwan, Iris E Eder

**Affiliations:** Department of Urology, Medical University of Innsbruck, 6020 Innsbruck, Austria; Lifespin GmbH, 93053 Regensburg, Germany; Department of Urology, Medical University of Innsbruck, 6020 Innsbruck, Austria; Department of Urology, Medical University of Innsbruck, 6020 Innsbruck, Austria; Department of Urology, Medical University of Innsbruck, 6020 Innsbruck, Austria; Department of Urology, Medical University of Innsbruck, 6020 Innsbruck, Austria; Department of Internal Medicine IV Nephrology and Hypertension, Medical University of Innsbruck, 6020 Innsbruck, Austria; Lifespin GmbH, 93053 Regensburg, Germany; Lifespin GmbH, 93053 Regensburg, Germany; Department of Urology, Medical University of Innsbruck, 6020 Innsbruck, Austria; Lifespin GmbH, 93053 Regensburg, Germany; Department of Urology, Medical University of Innsbruck, 6020 Innsbruck, Austria

**Keywords:** prostate cancer, clinically significant, prostate biopsy, tumor metabolism, urea cycle, NMR

## Abstract

**Context:**

Despite clinical suspicion of prostate cancer (PCa), 20% to 25% of patients exhibit a tumor-negative biopsy result.

**Objective:**

This work aimed to assess the serum metabolic profile of clinically significant (cs) compared to clinically insignificant (ci) PCa or benign (Be) patients.

**Methods:**

A total of 1078 serum samples were analyzed. Nuclear magnetic resonance (NMR) spectroscopy was used to quantify 73 metabolites; random forest was used for the model algorithm.

**Results:**

We identified a 22-metabolite panel, which discriminated csPCa (International Society of Urological Pathology [ISUP] 2-5, n = 328) from ciPCa (ISUP 1, n = 101) and Be patients (negative biopsy, n = 649) with a higher performance when combined with the standard clinical parameters age, prostate-specific antigen (PSA), and percentage free PSA (%fPSA) (area under the curve [AUC] 0.84) than the clinical parameters alone (AUC 0.73). Our study further revealed significant dysregulations of the urea cycle and the choline pathway along with changes in tricarboxylic acid cycle, cholesterol metabolism, and a significant increase of the inflammation marker glycoprotein acetyls B in csPCa patients. In particular, ornithine and dimethylglycine were the 2 most important features to discriminate csPCa from Be + ciPCa with significantly higher ornithine and lower dimethylglycine levels in patients with csPCa (ornithine: 63.7 ± 26.5 µmol/L, dimethylglycine: 12.6 ± 6.3 µmol/L; *P* < .001) compared to Be + ciPCa patients (ornithine: 50.3 ± 31.6 µmol/L, dimethylglycine: 14.9 ± 7.7 µmol/L).

**Conclusion:**

This study discovered a 22-metabolite panel to discriminate patients with csPCa from Be + ciPCa patients when combined with age, PSA, and %fPSA. It may therefore be used as a supportive biomarker to reduce the number of unnecessary biopsies and also to identify novel therapeutic targets in the future.

In 2022, prostate cancer (PCa) constituted 27% of all newly diagnosed cancers, making it the most prevalent cancer among men (1). Despite extensive efforts in biomarker research, prostate-specific antigen (PSA) remains the primary tool for early PCa detection. Since 2018, European Association of Urology guidelines recommended additionally performing a multiparametric magnetic resonance imaging (mpMRI) before biopsy for patients with elevated PSA levels. Moreover, a combined approach of mpMRI-directed targeted biopsy and systematic biopsy for patients with a Prostate Imaging Reporting and Data System (PI-RADS) score of 3 or greater is suggested (1). The biopsied tissue is then histopathologically assessed using the International Society of Urological Pathology (ISUP) classification. Patients are then further classified into men with clinically insignificant PCa (ciPCa, ISUP 1) and men with clinically significant PCa (csPCa, ISUP 2-5). This discrimination between ciPCa and csPCa is important with regard to a 15-year analysis, which recently showed that the cancer-specific survival of patients with ciPCa is high, irrespective of whether these patients are actively treated or only monitored (2). It has therefore been recommended to treat only patients with csPCa. Importantly, approximately 20% to 25% of patients receive a histologically negative biopsy result (3). Hence, bearing in mind that biopsy—either performed transperineally or transrectally—is invasive and potentially harmful, carrying risks such as bleeding or sepsis (4), there is an unmet medical need for the identification of reliable biomarkers to better identify benign (Be) patients and patients with ciPCa to avoid unnecessary biopsies.

Given the strong association between metabolic reprogramming and PCa, there has been growing interest in analyzing circulating metabolites to identify patients at risk of cs disease and thereby help to decrease the number of unnecessary biopsies (5). The analysis of circulating metabolites, which represent the end points of molecular pathways, is considered a highly promising strategy to decipher novel biomarkers. There have been a number of studies to identify novel metabolite profiles with potential usability in PCa diagnosis (6). In this study, we used nuclear magnetic resonance (NMR) spectroscopy, which is a highly reproducible, quantitative, and fast method without the need of complex sample preprocessing (7), to analyze a large retrospective cohort of 1078 serum samples from men who underwent biopsy due to suspicious PSA levels without any history of PCa (8). In brief, we identified a 22-metabolite panel that significantly discriminates patients with csPCa (ISUP 2-5) from Be + ciPCa patients.

## Material and Methods

### Study Design and Patient Characteristics

This study was approved by the ethics committee of the Medical University of Innsbruck (approval No. 1329_2021). Written informed consent was obtained from all participants. We used 1078 biobanked serum samples, which were collected from men with an age-related suspicious PSA without any history of PCa (8) and who therefore underwent prostate biopsy at the Department of Urology of the Medical University Innsbruck between 2004 and 2021. Blood was drawn before biopsy. In this study, all patients with a cancer-positive biopsy subsequently underwent radical prostatectomy. ISUP grading was determined on radical prostatectomy specimens. With the aim of finding a metabolite panel that identifies csPCa patients, we categorized all patients into 2 groups: (1) Be + ciPCa consisting of 750 patients, who either had a negative biopsy (Be, n = 649) or a positive biopsy with ciPCa (ISUP 1, n = 101), and (2) csPCa comprising 328 patients with a positive biopsy ISUP 2-5. In all patients, a transrectal biopsy approach was chosen, which was either systematic and mpMRI targeted (n = 409), systematic and real-time elastography-based targeted (n = 639), or systematic (n = 27) (9). In 3 cases, biopsy regimen was not specified. PSA and percentage free PSA (%fPSA) were determined at the time of serum sample draw. All patients’ demographic clinical characteristics are summarized in Table 1.

**Table 1.** Patients’ demographic clinical characteristics

	Be (total n = 649)		ciPCa (total n = 101)		PCa (total n = 328)	
	Mean (SD, IQR)	n (% of total n)	Mean (SD, IQR)	n (% of total n)	Mean (SD, IQR)	n (% of total n)
Age, y	58.6 (8.1, 12.0)	649 (100.0)	60.5 (7.7, 8.5)	101 (100.0)	62.0 (7.1, 10.0)	328 (100.0)
PSA, ng/mL	6.1 (7.0, 3.7)	649 (100.0)	5.5 (2.9, 4.0)	101 (100.0)	8.5 (8.4, 5.0)	328 (100.0)
%fPSA, %	18.8 (8.3, 9.3)	640 (98.6)	17.8 (7.0, 7.8)	101 (100.0)	14.1 (7.2, 8.1)	316 (96.3)
Prostate volume, mL	52.9 (25.5, 29.0)	582 (89.7)	48.4 (23.4, 24.0)	69 (68.3)	39.6 (18.1, 19.0)	209 (63.7)
PSA density, ng/mL/mL	0.12 (0.09, 0.06)	582 (89.7)	0.13 (0.07, 0.09)	69 (68.3)	0.24 (0.29, 0.17)	209 (63.7)
PI-RADS		143 (22.0)		38 (37.6)		134 (40.9)
1		0		0		0
2		13 (2.0)		0		4 (1.2)
3		26 (4.0)		6 (5.9)		4 (1.2)
4		95 (14.6)		27 (26.7)		94 (28.7)
5		9 (1.4)		5 (5.0)		32 (9.8)
Prostate biopsy		649 (100.0)		101 (100.0)		328 (100.0)
Systematic		22 (3.4)		0		5 (1.5)
Systematic + RTE-targeted		394 (60.7)		60 (59.4)		185 (54.4)
Systematic + mpMRI-targeted		231 (35.6)		41 (40.6)		137 (41.8)
Not specified		2 (0.3)		0		1 (0.3)
DRE		547 (84.3)		51 (50.5)		181 (55.2)
Non suspicious		526 (81.0)		48 (47.5)		158 (48.2)
Tumor suspicious		21 (3.2)		3 (3.0)		23 (7.0)
ISUP GG		0		101 (100.0)		328 (100.0)
1		0		101 (100.0)		0
2		0		0		221 (67.4)
3		0		0		60 (18.3)
4		0		0		16 (4.9)
5		0		0		31 (9.4)

	Mean (range)	Mean (range)	Mean (range)
No. of biopsy cores	14.4 (3-19)	14.8 (12-15)	14.6 (10-18)

Due to the retrospective design of this study, not all values were available for all patients.

Abbreviations: Be, benign; ciPCa, clinically insignificant prostate cancer; csPCa, clinically significant prostate cancer; DRE, digital rectal examination; %fPSA, % free PSA; GG, grade group; IQR, interquartile range; ISUP, International Society of Urological Pathology; mpMRI, multiparametric magnetic resonance imaging; PI-RADS, Prostate Imaging Reporting and Data System; PSA, prostate-specific antigen; RTE-targeted, real-time elastography targeted.

### Targeted Metabolite Analysis by Nuclear Magnetic Resonance Spectroscopy

Serum samples were isolated from whole blood by centrifugation and frozen at −80 °C until NMR analysis. Time from sample withdrawal until biopsy was 365 days or less. Before measurement, 350 µL serum were mixed with an aqueous buffer containing 0.1 g/L NaN_3_, 0.067 mol/L Na_2_HPO_4_, 0.033 mol/L NaH_2_PO_4_ (pH = 7.15 ± 0.05), 5% deuterium oxide (D_2_O) as field-lock substance. Pyrazine (6 mM) was used as an internal standard for quantification. NMR measurement was performed on a Bruker AVANCE NEO 600-MHz spectrometer. Measuring method was 1D 1H noesygppr1d_d20, NS = 16, T = 310 K. Measuring time per sample was 6.5 minutes. After passing routine quality, all measured spectra were Fourier-transformed using TopSpin software (version 4.0, Bruker Biospin), automatically phased (apk0.noe), and subjected to baseline correction (absn). Analysis was performed using the proprietary Lifespin Profiler software (version 1.4_Blood) by generating a quantitative metabolite list of 73 metabolites, where the concentration of each metabolite was given in µmol/L. Only “spectral shape parameter (ssp) lipids,” which decode NMR-detectable cholesterol in lipoproteins, and glycoprotein acetyls B (GlycB) were given in arbitrary units (au). The 73 analyzed metabolites were selected according to their intrinsic presence in blood serum samples at a concentration above the detection limit of the NMR method (∼1 µM, metabolite dependent). This includes amino acids and derivatives, organic acids, organic amines and amides, sugars, alcohols, ketone bodies, lipids, and other small metabolites. Quantification was based on a concentration adjustment against an internal standard without additional normalization.

### Statistics

In each serum sample 73 metabolites were quantified by NMR spectroscopy. Differential comparison of each detected metabolite between the groups was performed using the Wilcoxon-Mann-Whitney *U* test. Resulting *P* values were corrected for multiple testing (false discovery rate correction was made with respect to all 73 metabolites tested). The corrected *P* values were translated into * notation as follows: *** indicates *P* less than or equal to .001; ** indicates *P* less than or equal to .01; and * indicates *P* less than or equal to .05. Metabolite concentrations are expressed as box plots (1-99th percentile and median). Effect sizes are indicated by Cohen's *d*, which was calculated by dividing the difference of the means of the respective groups by their pooled SD and taking the absolute value. Considering absolute Cohen’s *d* values, effects were interpreted as small with a Cohen’s *d* value less than or equal to 0.5, moderate with a Cohen’s *d* value of 0.5 to 0.8, and large with a Cohen’s *d* value greater than or equal to 0.8. A negative Cohen's *d* value implies that the mean of the second group is higher than the mean of the first group.

### Model Building

To create a prediction model for csPCa, we split our data set into a training set (70% of the data were used) and a validation set (30%), respectively. For the distribution of the groups into the training and the validation set, the complete data set was split 70/30, while ensuring that the two individual groups were also split 70/30. For this, we performed random splits on both individual groups separately to ensure that both individual groups were equally split into a 70% test set and 30% validation set. Then, we performed a random forest (RF) algorithm to evaluate the performance of all 73 analyzed metabolites as well as of the 22-metabolite panel, which differentiates significantly between the groups. Twenty-one patients (Be n = 9, csPCa n = 12) were excluded from the analysis using the clinical parameters because the %fPSA values were not available. To assess the discrimination power of the model, we used receiver operating characteristics (ROC) analysis by plotting sensitivity vs (1-specificity) and calculated the area under the receiver operating curve (AUC). Variable importance was depicted as mean decrease Gini for each metabolite.

## Results

### Metabolic Alterations in Patients With Clinically Significant Prostate Cancer Compared to Benign + Clinically Insignificant Prostate Cancer Patients

**Figure 1.**
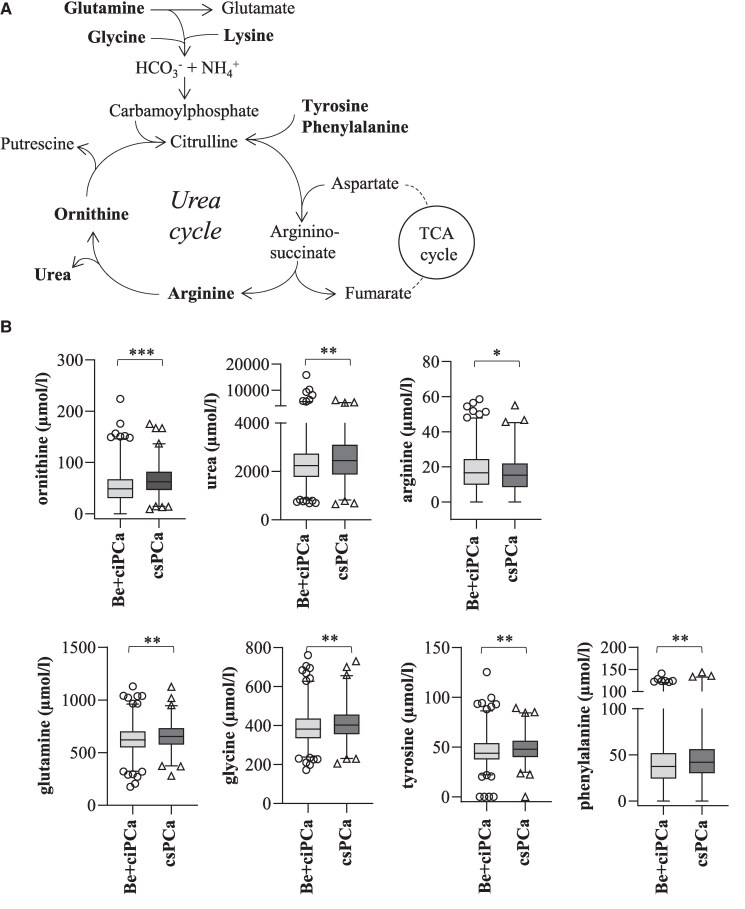
(A) Schematic illustration of the urea cycle showing its main metabolites and the link to the tricarboxylic acid (TCA) cycle. Metabolites significantly upregulated and downregulated in clinically significant prostate cancer (csPCa) patients are highlighted in bold letters. (B) Comparison of metabolite concentrations between benign (Be) + clinically insignificant prostate cancer (ciPCa) (n = 750), consisting of 649 men with negative biopsy (Be) and 101 patients with ciPCa, respectively, and csPCa patients (n = 328). Concentrations are given in µmol/L and expressed as box plots with whiskers (1-99th percentiles). Statistical analysis was performed with Wilcoxon-Mann-Whitney *U* test with adjusted *P* value: ***less than .001; **less than .01; *less than .05.

NMR analysis of 73 detected metabolites discovered a panel of 22 metabolites that were significantly upregulated or downregulated in patients with csPCa compared to Be + ciPCa patients (Table 2). Among these significantly altered 22 metabolites, ornithine, a key metabolite of the urea cycle (Fig. 1A), emerged as one of the top regulated features reflected by a Cohen’s *d* of −0.44. Serum ornithine levels were significantly higher in patients with csPCa (63.7 ± 26.5 µmol/L) compared to Be + ciPCa patients (50.3 ± 31.6 µmol/L) (*P* ≤ .001; Fig. 1B). Moreover, two other urea cycle metabolites, urea (*P* ≤ .01) and arginine (*P* ≤ .05), were significantly altered in serum samples of csPCa patients. Whereas urea was upregulated, arginine was downregulated in serum samples of csPCa compared to Be + ciPCa patients (see Fig. 1B). Correspondingly, the amino acids glutamine, glycine, tyrosine, and phenylalanine, which fuel the urea cycle, were statistically significantly (*P* ≤ .01, *P* ≤ .01, *P* ≤ .01, and *P* ≤ .01, respectively) upregulated in serum samples of csPCa patients compared to Be + ciPCa patients, highlighting the essential role of urea cycle dysregulation in PCa.

**Table 2.** Metabolites significantly increased or decreased in serum samples of patients with clinically significant prostate cancer compared to benign + clinically insignificant prostate cancer patients

Metabolite	Mean serum concentration ± SD, µmol/L		Corr. *P*	Cohen *d*
	Be + ciPCa	csPCa		
Ornithine	50.3 ± 31.6	63.7 ± 26.5	*^a^*	−0.44
Dimethylglycine	14.9 ± 7.7	12.6 ± 6.3	*^a^*	0.33
Lactic acid	2690.1 ± 1808.1	3020.4 ± 1624.3	*^a^*	−0.19
Propanol	7.2 ± 11.1	9.1 ± 11.3	*^a^*	−0.17
Ethanolamine	13.8 ± 9.2	15.6 ± 8.2	*^a^*	−0.21
Urea	2359.2 ± 1041.7	2555.3 ± 912.9	*^b^*	−0.20
Phenylalanine	39.1 ± 26.1	46.3 ± 26.2	*^b^*	−0.28
Glycine	391.1 ± 83.1	408.5 ± 77.4	*^b^*	−0.21
Dimethylamine	4.5 ± 2.4	3.9 ± 1.9	*^b^*	0.26
Glutamine	627.2 ± 120.3	654.4 ± 121.6	*^b^*	−0.23
Tyrosine	46.3 ± 13.4	49.1 ± 12.9	*^b^*	−0.21
Citric acid	163.6 ± 123.2	199.2 ± 145.6	*^b^*	−0.27
Pyruvic acid	51.4 ± 55.7	43.5 ± 30.4	*^c^*	0.16
1,2-Propanediol	6.3 ± 31.6	4.3 ± 21.5	*^c^*	0.07
Formic acid	16.2 ± 7.1	15.1 ± 6.8	*^c^*	0.15
Choline	19.7 ± 10.9	21.7 ± 13.3	*^c^*	−0.17
Arginine	18.0 ± 10.8	16.1 ± 10.3	*^c^*	0.18
Lysine	64.5 ± 20.7	67.4 ± 20.0	*^c^*	−0.14
3-Hydroxybutyric acid	69.9 ± 131.4	91.2 ± 264.3	*^c^*	−0.12

	Mean serum concentration ± SD, au		
GlycB	541.7 ± 144.2	572.7 ± 149.2	*^b^*	−0.21
Ssp lipid-1 (HDL)	38.2 ± 7.8	36.5 ± 7.8	*^b^*	0.22
Ssp lipid-4 (VLDL)	29.6 ± 20.8	34.4 ± 24.6	*^c^*	−0.22

Statistical significance was determined using the Wilcoxon-Mann-Whitney test. Mean metabolite concentrations with SD are given in µmol/L or arbitrary units (au). *P* values are accompanied by the respective effect sizes expressed as Cohen *d*.

Abbreviations: Be, benign; ciPCa, clinically insignificant prostate cancer; Corr., corrected; cs, clinically significant; GlycB, glycoprotein acetyls B, HDL, high-density lipoprotein; ssp, spectral shape parameter; VLDL, very low-density lipoprotein.

Metabolites are sorted by *P* values, which are corrected for multiple testing and are indicated as follows:

*^a^P* less than or equal to .001.

*^b^P* less than or equal to .01.

*^c^P* less than or equal to .05.

**Figure 2.**
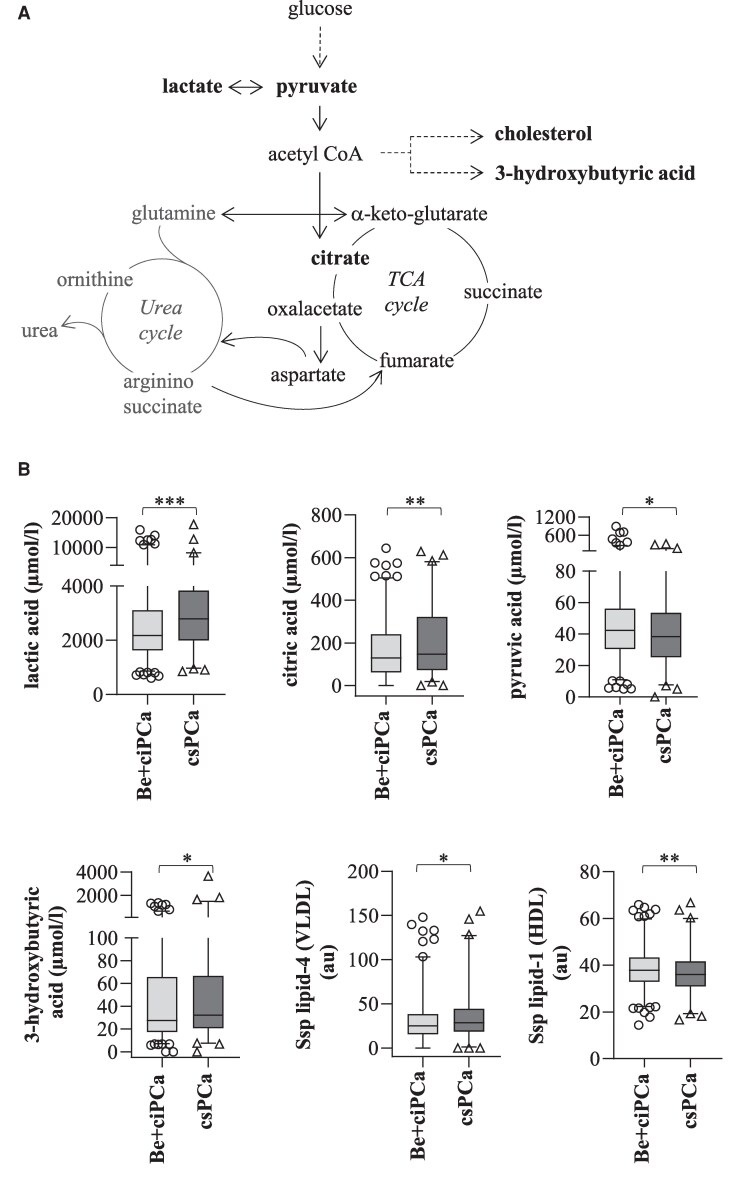
(A) Schematic illustration showing the link between urea cycle, tricarboxylic acid (TCA) cycle, and cholesterol metabolism with some of the main metabolites. Metabolites significantly upregulated or downregulated in clinically significant prostate cancer (csPCa) patients are highlighted in bold letters. (B) Metabolite concentrations in benign (Be) + clinically insignificant prostate cancer (ciPCa) (n = 750) and csPCa (n = 328). Concentrations are given in µmol/L or arbitrary units (au). Results were expressed as box plots with whiskers (1-99th percentiles). Statistical analysis was performed with Wilcoxon-Mann-Whitney *U* test with adjusted *P* value: ***less than .001; **less than .01; *less than .05.

Importantly, the metabolic profile of patients with csPCa also exhibited major changes in glucose and lipid metabolism, which are tightly linked to the urea cycle (Fig. 2A). Lactic acid was significantly higher in serum samples of csPCa patients (*P* ≤ .001) (Fig. 2B; Table 2) compared to Be + ciPCa patients. In addition, citric acid was significantly upregulated (*P* ≤ .01) whereas pyruvic acid was significantly downregulated (*P* ≤ .05) in patients with csPCa. Moreover, the ketone body 3-hydroxybutyrate was significantly higher (*P* ≤ .05) in csPCa patients compared to Be + ciPCa patients, which came along with significantly increased levels of ssp lipid-4 (very low-density lipoprotein [VLDL]) (*P* ≤ .05) and significantly decreased concentrations of ssp lipid-1 (high-density lipoprotein [HDL]) (*P* ≤ .01) (see Fig. 2B and Table 2), emphasizing the crucial role of cholesterol metabolism in PCa.

**Figure 3.**
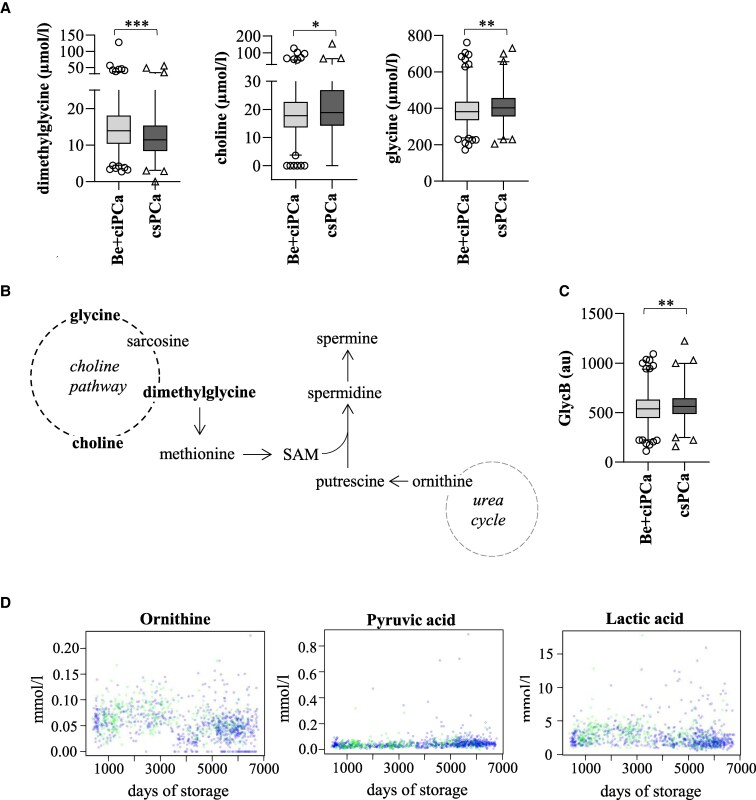
(A) Metabolite concentrations in benign (Be) + clinically insignificant prostate cancer (ciPCa) (n = 750) and clinically significant prostate cancer (csPCa) (n = 328) patients. (B) Schematic illustration of the choline pathway and some of its main metabolites with its link to the urea cycle and polyamine (putrescine, spermidine, spermine) synthesis through S-adenosyl methionine (SAM). Metabolites significantly altered in csPCa patients are highlighted in bold letters. (C) Comparison of glycoprotein acetyls B (Glyc B) levels between Be + ciPCa (n = 750) and csPCa (n = 328) patients. Concentrations are given in µmol/L or arbitrary units (au) and expressed as box plots with whiskers (1-99th percentiles). Statistical analysis was performed with Wilcoxon-Mann-Whitney *U* test with adjusted *P* value: ***less than .001; **less than .01; *less than .05. (D) Scatter plots showing the correlation between metabolite concentration in mmol/L for each sample (n = 1078) and storage time. Blue dots represent Be + ciPCa samples, green dots csPCa samples.

Among the top-ranked metabolites, we further found dimethylglycine to be significantly less presented in serum samples of patients with csPCa compared to Be + ciPCa (Fig. 3A). Dimethylglycine is a derivative of the amino acid glycine, which is converted to sarcosine within the choline oxidation pathway (10). The choline pathway is also tightly linked to the urea cycle via the synthesis of methionine and S-adenosyl methionine (SAM), thereby contributing to polyamine synthesis (Fig. 3B). Besides diminished dimethylglycine levels, choline and glycine were significantly increased in patients with csPCa, suggesting an intriguing role of the choline pathway in PCa.

Notably, our NMR-based metabolic profiling also revealed significantly increased levels of the inflammation marker GlycB in csPCa patients (572.7 ± 149.2 au) compared to Be + ciPCa patients (541.7 ± 144.5 au; *P* ≤ .01).

To reveal the integrity of our results with respect to sample storage, we next assessed the relationship between concentration and time of storage for the 22 significantly altered metabolites by computing the Spearman correlation coefficient. As shown in Fig. 3D, we found a weak correlation for ornithine (−0.32), pyruvic acid (0.29), and lactic acid (−0.26), implicating that the major metabolic changes in the urea cycle and choline metabolism were identified independently of sample storage time.

### 22-Metabolite Panel Increases the Predictive Value of Clinical Parameters to Discriminate Clinically Significant Prostate Cancer From Benign + Clinically Insignificant Prostate Cancer Patients

**Figure 4.**
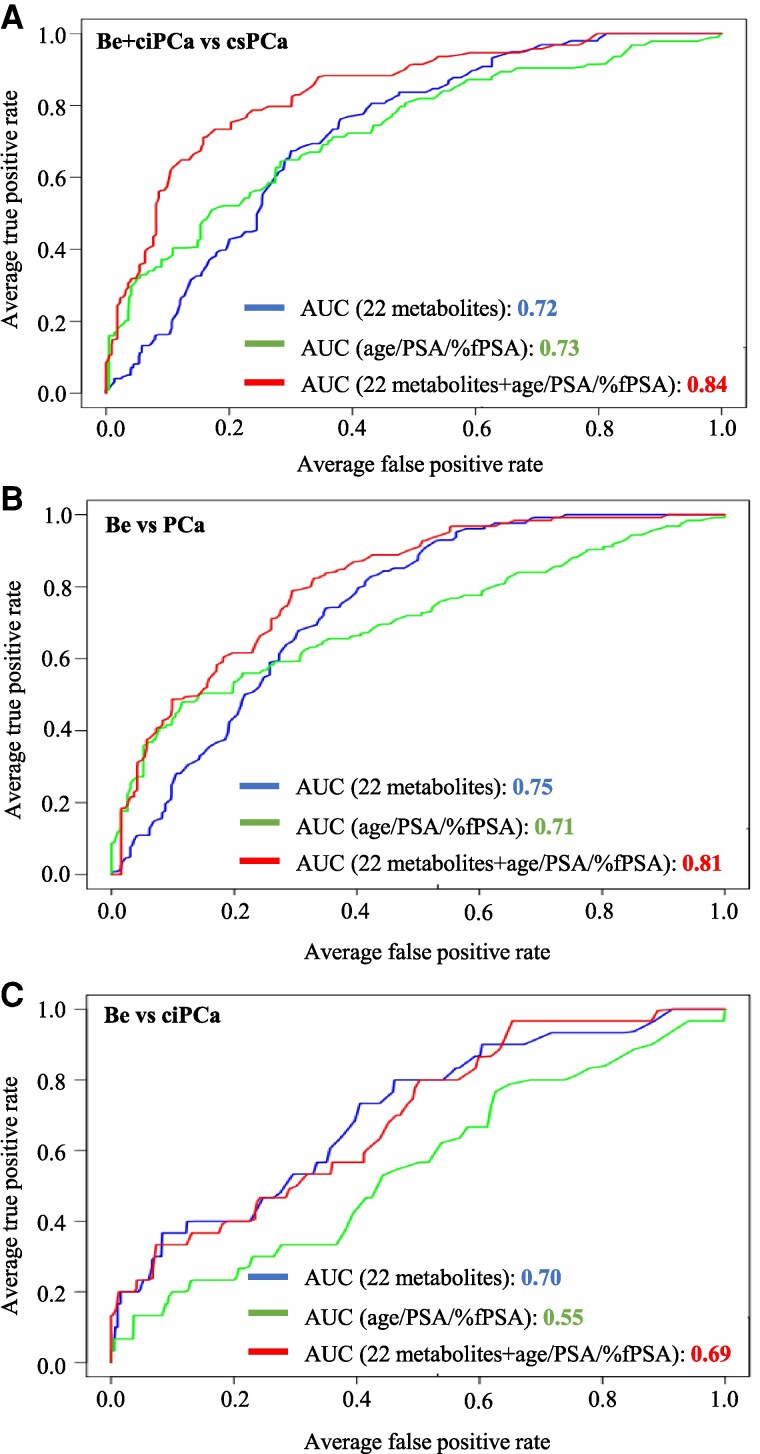
Receiver operating characteristic (ROC) analysis to assess the ability of the models to discriminate between patient groups. Random forest algorithm was performed by splitting the data set 70/30 into a training set and a validation set. Mean ROC curves were plotted for the nuclear magnetic resonance (NMR)-22-metabolite panel (22-metabolites, blue line), clinical parameters (age/PSA/%fPSA, green line), and a combination of both (22-metabolites + age/PSA/%fPSA, red line). (A) Benign (Be) + clinically insignificant prostate cancer (ciPCa) (n = 750) vs clinically significant prostate cancer (csPCa) (n = 328); (B) Be (n = 649) vs PCa (ISUP 1-5, n = 429); and (C) Be (n = 649) vs ciPCa (n = 101). The area under the curve (AUC) is given for each group comparison.

We next performed ROC analysis to assess the performance of the panel of 22 metabolites, which were significantly upregulated or downregulated in csPCa, either alone or in combination with the 3 clinical parameters age, PSA, and %fPSA to discriminate csPCa patients from Be + ciPCa patients. As shown in Fig. 4A, the 22-metabolite panel alone reached an AUC value of 0.72, thereby reaching a discrimination power that was similar to that of the clinical parameters age/PSA/%fPSA (AUC 0.73). When combining the clinical parameters (age/PSA/%fPSA) with the 22-metabolite panel, the AUC largely increased to 0.84. These data suggest that our 22-metabolite model represents a powerful supportive marker panel to improve the discrimination of csPCa from Be + ciPCa patients in a screening setting. Of note, similar results were obtained when using all 73 analyzed metabolites for the RF algorithm (73 metabolites AUC 0.73, age/PSA/%fPSA AUC 0.73, 73-metabolites + age/PSA/%fPSA AUC 0.80), emphasizing our selection of the 22-metabolite panel.

We next evaluated the discrimination power of the model for all PCa patients with a positive biopsy (ISUP 1-5) from patients with a negative biopsy (Be) (Fig. 4B). Notably, the discrimination power of our model for these two groups was very similar and reached an AUC of 0.81 when the 22 metabolites were combined with age/PSA/%fPSA. More important, our 22-metabolite panel clearly discriminated Be and ciPCa patients with an AUC of 0.70 (Fig. 4C), whereas the clinical parameters age/PSA/%fPSA—as expected—were not able to discriminate ciPCa from Be patients (AUC 0.55).

### Ornithine and Percentage Free Prostate-Specific Antigen Exhibit the Highest Feature Importance in Patients With Clinically Significant Prostate Cancer

**Figure 5.**
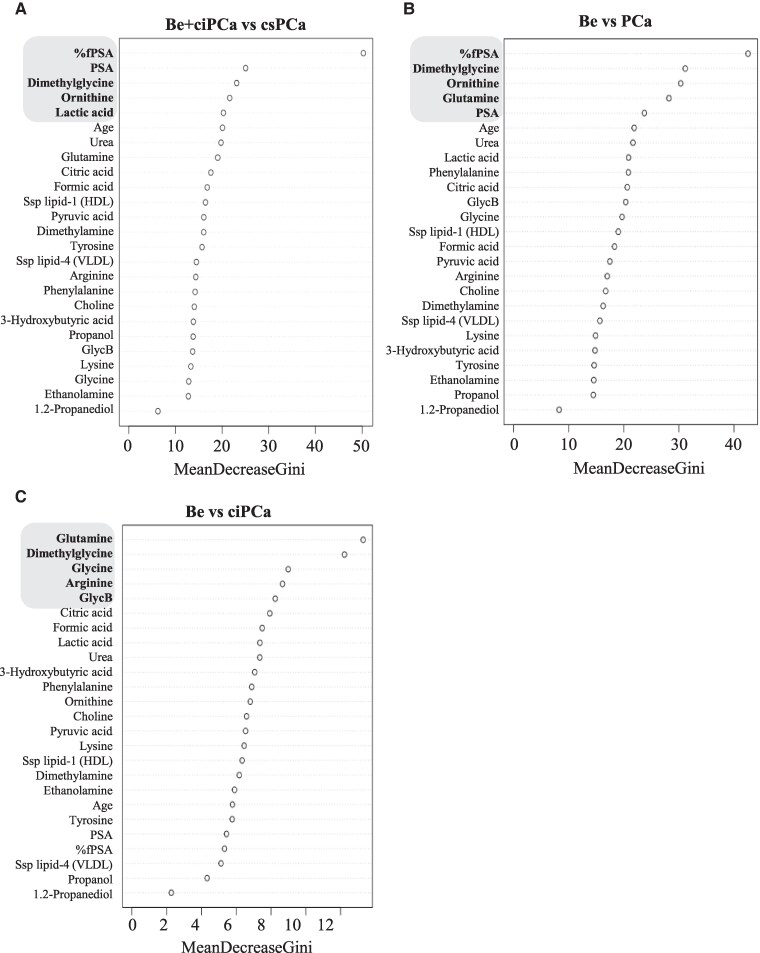
Random forest (RF) variable importance plots considering mean decrease Gini index. Mean decrease Gini indicates the importance of each feature for the prediction made by RF algorithm. Each point represents the mean decrease in Gini for each listed metabolite, sorted from top to the bottom, starting with the most important feature. The plots list the feature importance for (A) benign (Be) + clinically insignificant prostate cancer (ciPCa) (n = 750) vs clinically significant prostate cancer (csPCa) (n = 328); (B) Be (n = 649) vs PCa (ISUP 1-5, n = 429); and (C) Be (n = 649) vs ciPCa (n = 101). The 5 most important features are highlighted in gray.

We next investigated the importance of each metabolite for the prediction to discriminate the different patient groups by indicating the mean decrease in Gini. As shown in Fig. 5A, %fPSA, PSA, dimethylglycine, ornithine, and lactic acid were the top 5 ranked features with the highest importance to discriminate patients with csPCa from Be + ciPCa patients. Comparing all PCa patients (ISUP 1-5) with (Be) patients revealed %fPSA, dimethylglycine, ornithine, glutamine, and PSA as the 5 most important features (Fig. 5B). These findings support the importance of %fPSA and PSA as clinical biomarkers but also point out that metabolic dysregulation of the urea cycle and choline pathway are crucial events in PCa. Most notable, %fPSA and ornithine exhibited less importance in ciPCa patients, whereas the inflammatory marker GlycB emerged among the top 5 most important features to discriminate ciPCa from Be patients (Fig. 5C).

## Discussion

In this large, NMR spectroscopy-based metabolic study comprising 1078 serum samples, we identified a novel 22-metabolite panel, which was able to discriminate patients with csPCa from patients with ciPCa or Be patients, respectively. Combined with the standard biomarkers PSA, %fPSA, and age, this 22-metabolite panel reached an AUC of 0.84 and thereby outperformed the combination of the PCa biomarkers PSA, %fPSA, and age alone (AUC 0.73). Importantly, according to previously published findings, our model even reached a similar AUC value in the detection of csPCa as two other blood-based biomarker tests, the prostate health index and the Stockholm-3 test, which reached AUCs of 0.87 and 0.75, respectively (11, 12). It should be noted that the DRE status (tumor-suspicious vs nonsuspicious) was not included in the model, as not all values were available due to the retrospective nature of the study. Since the addition of DRE did not significantly improve the performance of the model in discriminating between Be + ciPCa or csPCa, we decided not to include these data in our manuscript. In addition, DRE alone showed poor performance in the PCa screening setting with a positive predictive value far below that of PSA alone. Therefore, DRE is no longer recommended for PCa screening in young men (13). These data suggest that combining our novel NMR-based 22-metabolite panel with the clinical biomarkers %fPSA, PSA, and age could be used for improved clinical decision-making in the future. Consequently, the analysis of this 22-metabolite panel could help to reduce the number of unnecessary biopsies. Of note, NMR spectroscopy has the advantage that samples can be analyzed in a few minutes without any costly preprocessing (7, 14), thereby paving the way for easy use in clinical diagnosis. We used a highly standardized and automated protocol for NMR analysis, which allows the measurement of large cohorts of samples in a timely, precise, and reproducible manner. Of note, each sample is digitized once the NMR spectrum is generated, so that the spectrum can be reanalyzed for additional metabolites/parameters at any time without the need to perform a new measurement.

In addition to a promising use of this 22-metabolite panel in diagnosis, our study further revealed important information on the metabolic phenotype of PCa. The RF algorithm revealed that besides the clinical markers %fPSA and PSA, dimethylglycine and ornithine were two of the most important features in discriminating patients with csPCa from patients with ciPCa or Be patients, indicating dysregulations of the urea cycle and the choline pathway. Ornithine is a central metabolite of the urea cycle (also called the ornithine cycle) and an important component for the synthesis of polyamines such as spermidine and spermine, which have been previously linked to PCa cell growth and proliferation (15). Besides ornithine, most of the main metabolites of the urea cycle (see Fig. 1A), namely glutamine, arginine, and urea itself were significantly altered in csPCa patients compared to patients with ciPCa or Be disease. In normal cells, the urea cycle is used to get rid of the toxic by-product ammonium that accumulates during amino acid catabolism. Cancer cells, on the contrary, are thought to produce less nitrogen waste and to salvage urea cycle intermediates for energy production by shunting carbon and nitrogen to biosynthetic routes instead (16). Matching with our own data, urea has previously been found by Zang et al (17) to discriminate men with Be prostates from PCa patients. Dysregulation of the urea cycle together with higher pyrimidine synthesis resulting in increased PCa cell proliferation has also been described by Lee et al (18). Concordantly, major enzymes of the urea cycle such as arginine-succinate synthase (ASS1), arginine-succinate lyase (ASL), and arginase (ARG) 1 and 2 are differentially expressed in PCa cells (19-21). There is also increasing evidence that PCa cells strongly rely on glutamine to support growth (22). Glutamine is used in the urea cycle for the production of citrulline, which is then further metabolized to ornithine and urea, or it is transformed to glutamate and then fuels the TCA cycle. Targeting glutamine metabolism with selective glutaminase inhibitors has recently been proven safe and tolerable in early-phase clinical studies (23). Overall, our data support the notion that urea cycle metabolites, in particular ornithine, play a crucial role in PCa.

In line with the accumulation of urea cycle metabolites, our study further revealed substantial alterations of TCA cycle-linked metabolites. Enhanced TCA cycle activity is known to support increased mitochondrial activity and to fuel de novo fatty acid synthesis in PCa cells (23). We found increased levels of citrate, phenylalanine, and tyrosine in serum samples of PCa patients. Of note, lactate was also among the top 5 most important features in serum samples of patients with csPCa. Though PCa has long been considered highly lipogenic instead of glycolytic like many other tumor types, there is now increasing evidence for lactate as another important metabolic fuel (24, 25). It has been assumed that lactate is highly produced by cells of the tumor microenvironment, including cancer-associated fibroblasts, thereby providing PCa cells additional energy for growth and proliferation (26). Probably as a consequence of the high lactate production, its precursor pyruvate was significantly decreased.

Rounding up a very distinctive phenotype of PCa, we detected significantly decreased HDL and increased VLDL cholesterol in csPCa patients compared to patients with ciPCa and Be patients. Cholesterol is an important precursor of androgens and represents an intriguing metabolite in PCa cells as previously reported by us and others (27-30). Moreover, fatty acids have long been considered an important energy source in PCa cells (31). Of interest, we also found increased production of the ketone body 3-hydroxybutyric acid in csPCa patients. Ketone bodies can be shunted into the TCA cycle or used for cholesterol synthesis (32) and thereby open up another source for the uncontrolled growth of cancer cells (27, 33, 34). In fact, ketone bodies were also found to be upregulated in blood, urine, and tissue PCa samples compared to those of healthy controls in previous studies (35). Of note, we have previously shown that cholesterol metabolism plays an important role in tumor progression and therapy resistance in PCa cells, which is significantly influenced by the interaction with cancer-associated fibroblasts (27).

This study further revealed a considerable dysregulation of the choline pathway. In particular, dimethylglycine was identified among the 5 top-ranked significantly altered metabolites. Dimethylglycine, a derivative of the amino acid glycine, which is further converted to sarcosine within the choline oxidation pathway (10), was significantly less present in csPCa compared to ciPCa and Be patients. These data are in accordance with previous findings by Perez-Rambla and colleagues (36), who determined decreased concentrations of dimethylglycine in the urine of PCa compared to patients with benign prostatic hyperplasia. Notably, our group of Be patients, which was defined as patients with a negative biopsy, inevitably also included patients with Be prostatic hyperplasia. This group of patients, however, was not further identified in our study because the aim was to find a marker that could discriminate Be from PCa patients to reduce the number of unnecessary biopsies. Choline and glycine, on the other hand, were significantly increased in patients with csPCa. Choline is involved in the synthesis of membrane phospholipids (37), whereas the amino acid glycine is involved in protein and nucleotide synthesis and methylation. Of note, sarcosine, which is an intermediate along the choline oxidation pathway and has been previously discussed as a potential marker for PCa (38), was not changed in our study cohort. By contrast to ornithine, dimethylglycine was also among the top 5 most important features in discriminating ciPCa patients from Be patients. Importantly, we also found that the inflammation marker GlycB (39) was significantly increased in patients with csPCa compared to patients with ciPCa or Be patients, indicating that metabolic changes in PCa are strongly associated with inflammation. In line with that, intraprostatic inflammation has been previously reported as an important hallmark of PCa (40).

### Limitations and Future Directions

One of the limitations of our study is the use of archived serum samples that have been collected over a period of 19 years. Long-term storage as well as inconsistencies in sample processing over time may influence metabolite stability. In our study we revealed only a weak correlation between sample storage time and metabolite concentrations, indicating that the major metabolic changes in urea cycle and choline metabolism, which were identified in this study, were highly independent of long-term storage. Nevertheless, investigating the influence of storage time and sample processing on metabolite concentrations is warranted and will be the subject of future investigations. In addition, diagnostic procedures in the course of the collection period were largely improved compared with the past. In fact, a considerable proportion of the samples in this retrospective study originate from the pre-mpMRI era and, therefore, mpMRI imaging could not be included in our discrimination model. We also cannot exclude that our group of Be patients contains some samples from patients with a false-negative biopsy result. Despite these limitations, however, the 22-metabolite panel together with the clinical parameters reached a high discrimination power. It should be noted that our preselection of the 22 statistically significant metabolites based on univariate *P* values can potentially decrease detected noise, but at the same time might also lead to a wrongful exclusion of small-effect metabolites. To evaluate this risk, we compared the results of the RF algorithm performed for all 73 analyzed metabolites with the results of the 22-metabolite panel, which showed very similar performance and variable importance. Another limitation is that statistical significance was calculated on the whole data set, which in consequence allowed for potential data leakage. However, as the analysis of the models on all metabolites showed very similar performance and variable importance, the authors judge that there was no overfitting. Due to the retrospective nature of our study, we also did not have access to clinical parameters such as diet, body mass index, family history, or medication. Based on our promising data showing that the 22-metabolite panel discriminates Be patients and ciPCa from patients with csPCa, we have now set up a prospective multicenter trial called PROSPIN at the Medical University of Innsbruck (study No. 20231113-3315) to validate the usability of our 22-metabolite panel for diagnostic purposes. In this prospective trial, we follow standardized conditions for sample collection, processing, and storage and collect a variety of additional clinical parameters, which—together with the metabolite profile—can be used to better characterize a PCa-specific metabolic phenotype.

### Conclusion

The 22-metabolite panel outlined in this study in combination with the clinical biomarkers age, PSA, and %fPSA showed robust capacity (AUC 0.84) in distinguishing csPCa from ciPCa or Be patients. Besides the strong potential of the 22-metabolite panel for improved diagnosis, our study identified a very characteristic phenotype in csPCa that showed substantial dysregulations of the urea cycle and the choline pathway together with changes in TCA cycle and cholesterol metabolism. These findings may not only help us to better understand the metabolic dysregulation in PCa but also to reduce the number of unnecessary and potentially harmful prostate biopsies in Be patients as well as in patients with ciPCa.

## Data Availability

Some data sets generated and/or analyzed during this study are not publicly available but are available from the corresponding author on reasonable request.

## Abbreviations

au: arbitrary units
AUC: area under the receiver operating curve
Be: benign
ciPCa: clinically insignificant prostate cancer
cs: clinically significant
DRE: digital rectal examination
GlycB: glycoprotein acetyls B
HDL: high-density lipoprotein
ISUP: International Society of Urological Pathology
mpMRI: multiparametric magnetic resonance imaging
NMR: nuclear magnetic resonance
PCa: prostate cancer
PI-RADS: Prostate Imaging Reporting and Data System
PSA: prostate-specific antigen
RF: random forest
RTE-targeted: real-time elastography targeted
ssp: spectral shape parameter
TCA: tricarboxylic acid
VLDL: very low-density lipoprotein
